# As Soon as You Taste It: Evidence for Sequential and Parallel Processing of Gustatory Information

**DOI:** 10.1523/ENEURO.0269-18.2018

**Published:** 2018-10-23

**Authors:** Raphael Wallroth, Kathrin Ohla

**Affiliations:** 1 Psychophysiology of Food Perception, German Institute of Human Nutrition Potsdam-Rehbruecke, Nuthetal 14558, Germany; 2 NutriAct – Competence Cluster Nutrition Research Berlin-Potsdam, Nuthetal 14558, Germany; 3 Cognitive Neuroscience, (INM-3), Institute of Neuroscience and Medicine, Research Center Jülich, Jülich 52425, Germany

**Keywords:** detection, discrimination, EEG, gustation, MVPA, taste

## Abstract

The quick and reliable detection and identification of a tastant in the mouth regulate nutrient uptake and toxin expulsion. Consistent with the pivotal role of the gustatory system, taste category information (e.g., sweet, salty) is represented during the earliest phase of the taste-evoked cortical response (Crouzet et al., 2015), and different tastes are perceived and responded to within only a few hundred milliseconds, in rodents (Perez et al., 2013) and humans (Bujas, 1935). Currently, it is unknown whether taste detection and discrimination are sequential or parallel processes, i.e., whether you know what it is as soon as you taste it. To investigate the sequence of processing steps involved in taste perceptual decisions, participants tasted sour, salty, bitter, and sweet solutions and performed a taste-detection and a taste-discrimination task. We measured response times (RTs) and 64-channel scalp electrophysiological recordings and tested the link between the timing of behavioral decisions and the timing of neural taste representations determined with multivariate pattern analyses. Irrespective of taste and task, neural decoding onset and behavioral RTs were strongly related, demonstrating that differences between taste judgments are reflected early during chemosensory encoding. Neural and behavioral detection times were faster for the iso-hedonic salty and sour tastes than their discrimination time. No such latency difference was observed for sweet and bitter, which differ hedonically. Together, these results indicate that the human gustatory system detects a taste faster than it discriminates between tastes, yet hedonic computations may run in parallel (Perez et al., 2013) and facilitate taste identification.

## Significance Statement

Perception and action reflects the culmination of multiple processing stages. In gustation, these stages and their sequence are understudied. We show strong correspondences between neural decoding onset and behavioral response times, demonstrating that differences between taste judgments are reflected early during chemosensory encoding, rather than resulting from higher-level cognitive processing. Moreover, we find that the processing sequence of detection and discrimination varies with taste contrast: detection precedes discrimination for sour-salty while both processes occur without time lag for bitter-sweet, which vary in hedonics. We suggest that hedonic features are processed in parallel to purely sensory computations with the potential to facilitate stimulus identification in taste perception, supporting the concept of a flexible sequence of gustatory coding states.

## Introduction

The innate ability to discriminate between basic taste categories (Cowart, 1981; Steiner et al., 2001) reflects the ecological imperative of the mammalian sense of taste and underlines its role in nutrient sensing and the avoidance of harmful substances. Indeed, sweet taste indicates the availability of carbohydrates, salty taste allows electrolyte detection, umami taste serves protein recognition, and sour and bitter tastes alert us to acids and potentially harmful substances like alkaloids, respectively (Breslin, 2013).

Each taste category is detected by specific receptors, mostly on the tongue (Roper and Chaudhari, 2017), and taste-specific information is transduced to the brainstem, eventually arriving at dissociable cortical representations (Katz et al., 2002; Schoenfeld et al., 2004; Pavao et al., 2014; Crouzet et al., 2015; Wallroth et al., 2018). Despite detailed descriptions of peripheral and central sites of gustatory information processing, the emergence of the taste processing cascade, such as the detection of and discrimination between tastes, is not yet understood.

Early investigations of human taste behavior demonstrated that tastes can be detected within only 200 ms (Lester and Halpern, 1979; Yamamoto and Kawamura, 1981), and that more complex taste judgments such as identification and discrimination take 100–200 ms longer (for an overview, see Halpern, 1986). Interestingly, Kuznicki and Turner (1986) hypothesized that taste discrimination times are intimately linked with the time required to detect individual tastants (termed “time criterion strategy”). Accordingly, during the discrimination of tastes with different detection latencies, the faster taste serves as a cue that triggers the response, which results in an apparent speed-up of the discriminatory decision for the slower taste. Contrarily, when tastes with similar detection latencies are to be discriminated, the absence of such a response cue slows the discriminatory decision considerably as compared to their individual detection times (Kuznicki and Turner, 1986).

Generally, differential timing between simple and more complex evaluations (e.g., detection of a taste or judging its intensity) has been largely attributed to central processing, as neither correlations of the temporal properties of the taste periphery nor chemical properties of the tastants could account for the magnitude of the observed differences (Halpern, 1986; Kelling and Halpern, 1986). However, given that behavioral outputs reflect the culmination of several processing stages, prior work was unable to address whether the observed timing differences between taste judgments, particularly taste detection and identification, are a consequence of early central processing associated with chemosensory encoding or later central processing associated with higher-level cognition, such as decision making. To this end, investigating the occurrence of taste-related responses in ongoing neural activity (e.g., via electrophysiological recordings) provides an ideal tool to address whether attentional modulation affects early sensory processing or higher-level cognition such as memory, response selection, etc. (Luck and Hillyard, 1999). So far, our mechanistic understanding of the taste processing sequence is based on rodents, where single neuron recordings in the gustatory cortex revealed separable stages of taste-nonspecific action potential bursts, which likely represent oral somatosensation, and more complex, taste-specific responses (Katz et al., 2001; Baez-Santiago et al., 2016), although these findings cannot be readily transferred to humans given differences between species and experimental protocols. Further findings suggest that gustatory responses are not represented by stationary sensory codes but are subject to contextual modulations such as attention and expectation (Fontanini and Katz, 2006, 2009; Samuelsen et al., 2012).

In comparison with other sensory systems, the olfactory sense may afford the most relatable insights, as major perceptual computations conclude within a time frame akin to the gustatory sense (cf. Crouzet et al., 2015; Jiang et al., 2017), with a temporal advantage for detection over discrimination performance of comparable magnitude (∼200 ms; cf. Halpern, 1986; Olofsson et al., 2013). In olfaction, response-time data suggest a cascade with distinct processing stages for detection, identification, and edibility, which unfold in a causal, sequential manner, while valence computations may also run, at least in part, in parallel to identification (Olofsson et al., 2013). In contrast, detection and categorization of visual objects (such as “bird” or “car”) may in fact occur simultaneously (Grill-Spector and Kanwisher, 2005), although it has also been suggested that detection and identification are not intrinsically linked but rather are contingent on a variety of task factors (Mack et al., 2008).

Here, we investigated the processing sequence of two distinct taste judgments: detection and discrimination. Specifically, we tested whether temporal differences between taste detection and discrimination are already reflected at the early stages of sensory encoding or only manifest during later stages related to higher-level cognitive processing, using multivariate pattern analysis of gustatory electroencephalography (EEG) and psychomotor response times (RTs).

## Materials and Methods

### Participants

Twenty-one healthy and normal-weight individuals participated in the experiment and received monetary compensation or class credits. Exclusion criteria were heavy smoking, pregnancy, impaired sense of taste, hearing aid, and past or current neurologic or psychological disorders; the information was self-report based. One subject was excluded from all analyses due to technical difficulties during data collection. One participant completed only the EEG part and did not participate in the rating procedure; we kept this partial data set. Accordingly, data from 20 participants, 16 women, 18–34 years old (mean age 25.27 ± 4.04 SD; mean BMI 21.82 ± 2.66 SD), are reported for the EEG and behavioral data, and data from 19 participants, 15 women, 18–34 years old (mean age 25.40 ± 4.10 SD; mean BMI 21.97 ± 2.64 SD), are reported for the ratings. The study conformed to the revised version of the Declaration of Helsinki and was approved by the ethics board of the German Psychological Society. Participants provided written informed consent before participation.

### Materials

Four solutions with a clear taste were presented to participants: 0.684 M sodium chloride (salty; local supermarket, REWE, Köln, >97% purity), 0.052 M citric acid (sour; SAFC, CAS#77-92-9, Sigma-Aldrich, Inc.), 0.003 M quinine monohydrate (bitter; CAS#207671-44-1, Sigma-Aldrich, Inc.), and 0.075 M Splenda (sweet; Tate & Lyle*). Solutions were prepared daily by dissolving the chemical in distilled water.

Taste and rinse solutions were delivered with the GU002 gustometer (Burghart Messtechnik GmbH), which stores solutions in separate bottles that each supply a syringe pump with a check valve (Iannilli et al., 2015). From there, solutions are transported via separate, 5-m-long Teflon tubes to a manifold outlet where they mount together with compressed air to a spray nozzle that atomizes the liquids to an even spray. The spray nozzle is positioned 1–1.5 cm above the slightly extended tongue so that the spray covers a large area of the anterior, slightly extended tongue’s surface. All tubes ran inside a hose filled with water at 38°C until the manifold to keep the solutions at a constant temperature and to minimize any thermal sensations. During the experiment, the participant comfortably leaned against a forehead rest, which stabilized the head and held the spray nozzle in place. In this position, liquids were applied to the slightly extended tongue and not swallowed but collected in a bowl underneath the chin. The position was monitored online via camera to monitor positioning of the tongue and movements.

The gustometer was set to apply regular, distinct spray pulses. During each pulse, 70 µl of liquid were dispensed during 100 ms; this period was followed by a pause of 200 ms, which served to separate consecutive spray pulses. Each taste stimulus consisted of three such pulses and amounted to a bolus of 210 µl delivered over a period of 900 ms (flow rate: 233 µl/s). The timing and flow rate were optimized to minimize mixing of individual spray pulses and to elicit the experience of a continuous flow of liquid to the tongue. The distinct spray pulses permit to embed a tastant in the “flow” of control or water stimuli without tactile cue. Notably, participants experience a tactile “pulsing” only for a few seconds until the lingual somatosensory system is habituated. During the development of this procedure, we determined the time required for lingual habituation; we measured the time to the abolishment of the lingual somatosensory steady-state response and confirmed our findings with verbal reports of numbing of the tongue. The steady-state response was abolished within <10 s. As a result, we present water pulses for at least 10 s at the beginning of each experimental block or experiment (Tzieropoulos et al., 2013; Crouzet et al., 2015). The time between the TTL pulses controlling the syringe plungers, which push the liquids through the tubes and the spray nozzle, until the aerosol reaches the tongue’s surface, was measured by the supplier for the experimental setting described here following a previously proposed conductivity measurement (Kelling and Halpern, 1986). It revealed a time lag of 36 ms (SD = 2 ms), which the stimulus onset in the EEG data were corrected for.

### Design

**Figure 1.**
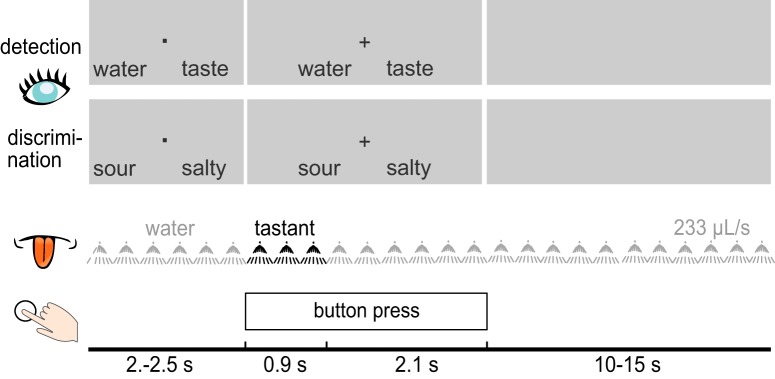
Schematic illustration of the experimental design during the detection and discrimination tasks. The first two rows portray examples of visual cues displayed to participants during detection and discrimination trials. During each trial, a liquid tastant (black) was embedded in a sequence of water pulses. Participants were to speededly respond by button press during both tasks.

Participants completed two forced choice RT tasks, which alternated block-wise and each repeated four times for a total of eight blocks (see Figure 1). In the “detection” task, participants were asked to decide whether they received a tastant (any of the four) or water, and to respond with the appropriate button press as quickly as possible. There were 160 tastant trials (40 per tastant) and 160 water trials, for a total of 320 detection trials. In the “discrimination” task, participants were asked to decide between two pairs of tastes. There were 160 discrimination trials in total (40 per tastant). The discrimination was performed for two pairs: salty versus sour and sweet versus bitter. The tastant pairs were selected based on three criteria: (1) same type of taste receptors: salty and sour taste are signaled via ion channels, and for sweet and bitter via G protein-coupled receptors (GPCRs), which convey information at different speeds (Pfaffmann, 1955); (2) similar behavioral response speed: taste detection responses are faster for salty and sour than for sweet and bitter (Yamamoto and Kawamura, 1981; Kuznicki and Turner, 1986), which, according to the time criterion hypothesis, would lead to the faster taste serving as a response cue in a discrimination; (3) similar cortical response latencies: similarly to reaction times, salty and sour evoked earlier cortical responses than sweet and bitter (Kobayakawa et al., 1999; Crouzet et al., 2015).

At the beginning of each trial, a fixation dot was displayed along with two answer options, with the option on the left corresponding to the leftmost button on the button box, and the option on the right corresponding to the rightmost button. The response mappings were pseudo-randomized across trials and equiprobable. A fixation cross replaced the fixation dot after 2–2.5 s to indicate that the gustatory stimulus (taste or water) was being administered, and that participants should respond with the respective button press. After 3 s, a gray screen was displayed until the next trial. The rinsing period between trials was 15 s for discrimination, and was shortened to 10s in the detection task, due to the inclusion of water trials. Rinsing started immediately after tastant presentation and continued until the next tastant. After the eight task blocks, participants completed a short evaluation block, in which each tastant was presented once more in pseudo-random order and participants were to rate intensity and pleasantness on a horizontal 101-point visual analog scale anchored with 0 (corresponding to no sensation) and 100 (extremely intense) and with −50 (extremely unpleasant) and 50 (extremely pleasant), respectively. The experiment lasted ∼120 min, including breaks.

### EEG data acquisition

Participants were seated in a sound-attenuated recording booth (Studiobox GmbH) with the gustometer positioned outside. The EEG was recorded with an activCHamp amplifier system (Brain Products GmbH) at a sampling rate of 500 Hz with analog 0.01-Hz high-pass and 200-Hz low-pass filters using PyCorder (Brain Vision LLC) with 64 Ag/AgCl active electrodes placed in an elastic cap according to the extended 10/10 system.

### EEG data preprocessing

The EEG data were processed offline using custom MATLAB- and Python-based scripts with functions from EEGLAB (Delorme and Makeig, 2004) and Autoreject (Jas et al., 2017), respectively. Data were first down-sampled to 200 Hz to improve the signal-to-noise ratio and computation speed. Slow drifts were corrected with linear de-trending and line noise (50 Hz) was removed with a set of multi-tapers over sliding time windows. The continuous data were then segmented into epochs spanning from −0.5 to 3 s relative to stimulus onset and Autoreject was applied to interpolate noisy channels within epochs. Next, an extended Infomax independent component analysis (ICA; Makeig et al., 1997) was computed to identify artifactual components with manual inspection guided by ADJUST (Mognon et al., 2011), which uses temporal and spatial characteristics of the independent components (ICs) to detect outliers. ICs representing common artifacts were subtracted from the data. The data were then re-referenced to the average of all electrodes. Finally, we applied zero-phase Hamming-windowed sinc finite impulse response filters (cutoff: −6 dB, maximum passband deviation: 0.2%, stopband attenuation: −53 dB) to isolate the frequency spectrum below 6 ± 2 Hz (order: 330) and above 0.5 ± 1 Hz (order: 660), and subsequently shortened the epochs to −.2 s to 1.5 s. The frequency cutoff was based on recent findings showing that taste quality information is encoded within the power and phase information of the δ and lower θ frequency bands (roughly up to 6 Hz; Hardikar et al., 2018; Wallroth et al., 2018; see also Pavao et al., 2014). Trials were then normalized by subtracting the average of each electrode’s baseline period (−200 ms to stimulus onset) before decoding analysis. No trials were excluded from the data.

### Descriptive EEG analysis

**Figure 2.**
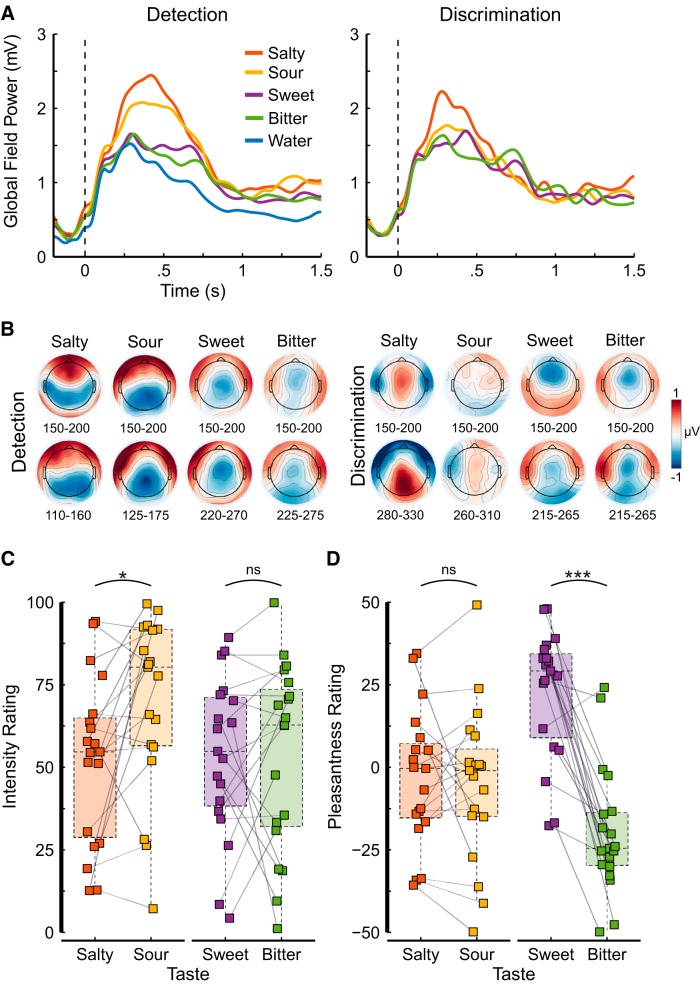
***A***, Signal strength quantified as the average GFP computed within-subjects as the standard deviation of the event-related potentials over 64 electrodes for each of the tastants and water over detection trials (left) and discrimination trials (right). Salty and sour tastants show a stronger signal than sweet and bitter tastants, but less strongly so for discrimination trials. Note that the onset of the liquid stimulation (for all tastes and for water) coincided with the presentation of the fixation cross, resulting in a clear GFP response for water as well. ***B***, Topographical voltage maps for each taste and task represent the grand-averaged mean over a 50-ms time window, early during processing (upper row) and surrounding the decoding onset (lower row) shown in Table 2 and Figure 3*C* relative to water. Intensity (0–100; ***C***) and pleasantness ratings (-50–50; ***D***) for the two tastant pairs, salty-sour and sweet-bitter. The colored squares show individual participant ratings, the gray lines between two squares indicate that these ratings were given by the same participant. Semitransparent and colored boxplots entail the ratings of all participants (*N* = 19); the horizontal dashed line within each box represents the median, the bottom and top of the box represent the first and third quartiles, respectively; whiskers show 1.5 times the interquartile range. The colors represent the taste. Significance is indicated above the plot area: ns *p* > 0.05; *p < 0.05; ***p < 0.001.

To quantify the strength of the electrophysiological signal for each experimental condition, we computed the global field power (GFP), a reference-free index of electric field strength, per task and taste. The GFP is a measure of variance (i.e., the average of the standard deviations of the event-related potentials at each of the 64 electrodes) and expresses how much electrical activity (averaged across participants) occurs in response to an event (Fig. 2*A*). To illustrate the electric field distributions, we computed topographical voltage maps for each taste and task. Each map represents the grand-averaged, mean voltage from 150 to 200 ms and 50 ms surrounding the mean decoding onset time relative to water (Fig. 2*B*). Difference maps were computed to remove the visual evoked response elicited by the display of the fixation cross.

### Decoding analysis

To determine the time point at which information related to detection and discrimination of tastes is represented at the single-trial level, we performed a time-resolved multivariate pattern analysis on the amplitudes of all 64 electrodes (MVPA; Kriegeskorte, 2011) embedded in a temporal generalization method (King and Dehaene, 2014). For each participant, the MVPA was implemented with multiple binary L2-regularized logistic regression classifiers (Fan et al., 2008). To mimic the behavioral tasks, four classifiers were trained to detect one of the tastants contrasted to water (using trials from the detection task), and two classifiers were trained to discriminate the two tastant pairs (salty-sour and sweet-bitter, using trials from the discrimination task). The procedure was implemented with a stratified leave-one-trial-out cross-validation (i.e., on every iteration, a trial of each taste is left out). Trials with incorrect behavioral responses were excluded from decoding.

Using the temporal generalization method, a taste-related activity pattern learned at one time point on the population level of trials (reflecting an average) is generalized backward and forward in time, given the time series of a single trial. The resulting classification performance reflects the correspondence between single and average trial activity across time. Unlike the common MVPA approach with pattern learning and testing performed exclusively at identical time points, this generalization approach is better suited to determine activity onsets at the single-trial level by fully taking into account the trial-to-trial variability of gustatory processing states (cf. Jones et al., 2007). Hence, trial-level taste-related activation patterns before or after the average taste response can still be detected.

To determine the onset of the taste-signal at the single-trial level, we used a searchlight approach in line with the “maximum cluster area” statistic (i.e., a predefined number of neighboring time-points exceed a statistical threshold; cf. Bullmore et al., 1999). Given that the sigmoid function of the logistic regression naturally quantifies the certainty with which a classifier makes its decision, we defined a classification as accurate when the correct choice was made with a certainty exceeding the 95% confidence interval of the binomial threshold (a common statistic in classification analysis because it adapts the chance level to the sample size; cf. Combrisson and Jerbi, 2015). Because the decisional certainty is strongly affected by the hyperparameter C (the regularization constant), with negligible influence on the overall performance, we fixed the parameter at C = 0.005, which essentially shrinks the standard deviation of the normal distribution of decision values (as compared to the default of C = 1) for more robust onset estimations. The cluster size is a free parameter which was defined as 50 ms of a stable pattern average (*x*-direction) and 100 ms of 95% successful generalization (*y*-direction). This cluster-asymmetry reflects our prioritization of stable estimates at the single-trial level over average pattern stability. The taste-signal onset was defined as the earliest generalization timepoint in the first cluster of significant decoding performance.

Notably, this type of temporal clustering is more liberal with respect to the adjustment for multiple null hypothesis testing than the alternative permutation-based approach (cf. Maris and Oostenveld, 2007). However, the latter (stricter) procedure is better suited to identify whether or not an effect is present, rather than when it first occurs. Given previous findings that taste qualities can be successfully decoded from EEG recordings (cf. Crouzet et al., 2015; Hardikar et al., 2018; Wallroth et al., 2018), our chief concern was to find an adjustment procedure which balances Type I and Type II error rates such that we would identify the taste-signal onset as accurately as possible (i.e., with a minimal number of false alarms but also as few misses of the true signal). To summarize, our present motivation was to explore exactly when a taste-signal emerges at the single-trial level, rather than to investigate whether a taste-signal occurs at all.

The classifier performance was summarized for grand-average visualization as the area under the receiver operating characteristic curve (AUC), and for the statistical analysis of the single-trial results the accuracy was defined as the percentage of trials for which an onset was determined successfully.

### Statistical analysis

Statistical analyses were performed with R (R Core Team, 2017). Ratings were analyzed using Student’s *t* tests to compare the tastes within a pair, sour with salty and sweet with bitter and the degree of pleasantness (positive, neutral, or negative) was tested using one-sample *t* tests against a null hypothesis of zero, with zero corresponding to neutral on the rating scale. For each of the dependent variables RT, accuracy, decoding onset, and decoding accuracy and for each taste pair (sour-salty or sweet-bitter), a two-way repeated measures ANOVA with the factors TASK (detection, discrimination) and TASTE were computed. Paired samples Student’s *t* tests of the difference between discrimination and detection were used to resolve TASTE v TASK interactions. One-sided Pearson correlations were computed of the difference values between detection and discrimination decoding onset and RTs to verify the correspondence between neural and behavioral effects. The α-level was a priori set to 0.05; for violations of sphericity Greenhouse–Geisser correction was applied to the degrees of freedom. We report uncorrected degrees of freedom and the absolute values of Cohen’s *d* effect size estimations.

## Results

### Ratings

Stimulus concentrations were chosen based on previous studies such that all tastants are clearly perceivable, that tastants within a pair were similarly intense, and that tastants were acceptable (Fig. 2*C*,*D*). Overall, all tastes were moderately intense (mean intensity range, 52.35–69.97; Fig. 2*A*). Bitter and sweet were iso-intense (*t*_(18)_ = 0.03, *p* = 0.978, *d* = 0.01); yet sour was more intense than salty (*t*_(18)_ = −2.83, *p* = 0.022, *d* = 0.43). As expected, salty and sour were neutral in pleasantness (*t* test against zero; salty: *t*_(18)_ = −0.67, *p* = 0.680, *d* = 0.22; sour: *t*_(18)_ = −0.92, *p* = 0.594, *d* = 0.30), and both were similarly pleasant (*t*_(18)_ = 0.41, *p* = 0.784, *d* = 0.05). Bitter and sweet, on the other hand, varied strongly in pleasantness (*t*_(18)_ = −7.13, *p* < 0.001, *d* = 0.99) such that bitter was clearly unpleasant (*t*_(18)_ = −4.44, *p* < 0.001, *d* = 1.44) and sweet was clearly pleasant (*t*_(18)_ = 5.00, *p* < 0.001, *d* = 1.62), which was to be expected (Fig. 2*D*).

### Behavioral data

**Figure 3.**
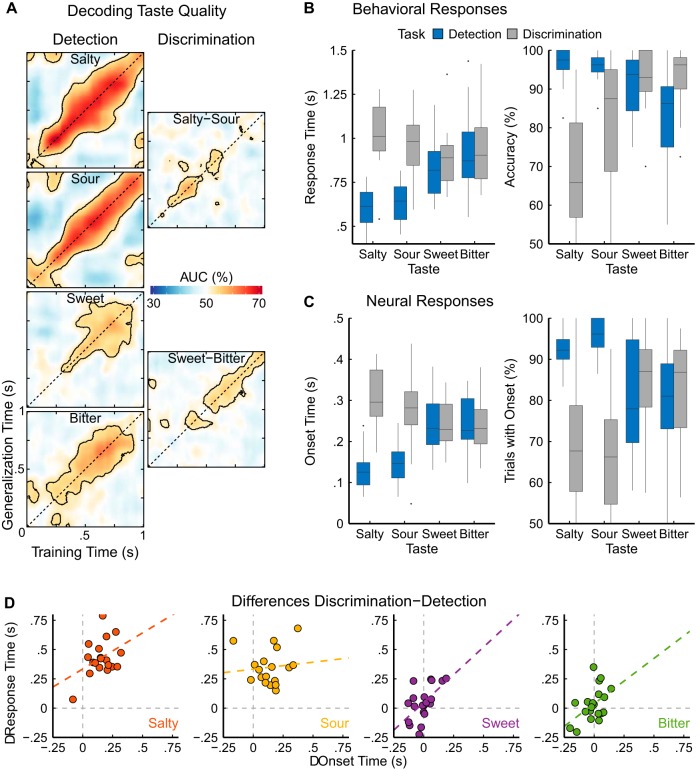
***A***, Average within-subject decoding generalization across time for each of the four tastes by task. Detection performance is obtained for the classification of a tastant against water (detection task trials); discrimination performance is obtained for the classification between two tastants (discrimination task trials). The diagonals of the matrices (identical training and testing time) correspond to the common decoding approach. The *x*-axis displays training times which represents the stability of an average taste pattern. The *y*-axis displays generalization or testing times which represents the emergence of the average pattern (*x*-axis) within individual trials. Warm colors reflect average performance increases as compared to chance level (50%), cold colors reflect decreases; black contour lines indicate statistical significance of the grand average as assessed via one-sided cluster-adjusted binomial tests (*p* < 0.05). Salty and sour show earlier and better detection performance than sweet and bitter, whereas discrimination performance is less pronounced than detection performance in either case. ***B***, Behavioral data of the button press RTs of correct responses and accuracy (average per participant, *N* = 20) color-coded for tasks (blue indicating detection trials, gray discrimination trials). The horizontal line in each boxplot represents the median, the bottom and top of the box represent the first and third quartiles, respectively; whiskers show 1.5 times the interquartile range, dots indicate outliers. Participants are faster and more accurate at detecting salty and sour than they are at discriminating the two tastants. Sweet and bitter show no difference in RTs but higher accuracy at discriminating the two as opposed to detection from water. ***C***, Neural data of onset times of above-chance performance (determined at the single-trial level; averaged per participant; *N* = 20 for sweet and bitter tastes, and *N* = 18 for salty and sour tastes) and of the accuracy indicating the percentage of trials for which such an onset was determinable (boxplot parameters as in ***B***). The neural findings correspond closely to the behavioral data in that salty and sour are classified faster and more accurately in detection trials. Sweet and bitter show no significant difference between the two tasks. ***D***, Correlations of the difference values between the average discrimination and detection neural onset times and button press RTs (each point in a graph represents one participant). Color-coded dashed lines represent linear regression models; horizontal and vertical gray dashed lines indicate the points of no difference between discrimination and detection latencies on the respective axis. The observed effects were significantly positively correlated for three of four tastes, such that an early neural difference (or lack thereof) corresponded to the same behavioral effect.

In line with the study design, statistical analyses were conducted separately for the taste pairs “sour-salty” and “sweet-bitter.” RTs and accuracy are summarized in Table 1 and shown in Figure 3*B*.

**Table 1.** Descriptive statistics of RTs and accuracies

	Detection				Discrimination			
	RT (ms)		Accuracy (%)		RT (ms)		Accuracy (%)	
Taste	M	SEM	M	SEM	M	SEM	M	SEM
Salty	609	24	96.1	1.1	1029	39	67.6	4.3
Sour	642	24	95.9	0.8	964	38	80.6	4.3
Bitter	905	51	81.9	3.2	938	45	93.3	1.7
Sweet	835	37	91.1	1.8	881	36	92.0	2.0
Water	906	38	95.6	1.0	-	-	-	-

RT = reaction time.

For the salty and sour contrast, detection RTs were significantly faster than discrimination RTs (*F*_(1,19)_ = 119.61, *p* < 0.001, η^2^ = 0.64), and RTs were similar for both tastes (*F*_(1,19)_ = 1.08, *p* = 0.310, η^2^ = 0.003). A task × taste interaction was observed (*F*_(1,19)_ = 18.70, *p* < 0.001, η^2^ = 0.03), and the comparison of the difference between detection and discrimination revealed that the effect was larger for salty than for sour (*t*_(19)_ = 4.32, *p* < 0.001, *d* = 0.45). Accuracy was significantly higher in the detection than in the discrimination task (*F*_(1,19)_ = 38.24, *p* < 0.001, η^2^ = 0.39) and also higher for sour than for salty (*F*_(1,19)_ = 6.91, *p* = 0.020, η^2^ = 0.05). Again, a task × taste interaction was observed (*F*_(1,19)_ = 6.26, *p* = 0.020, η^2^ = 0.06), and the comparison of the difference between detection and discrimination revealed that the effect was larger for salty than for sour (*t*_(19)_ = −2.50, *p* = 0.022, *d* = 0.79).

For the sweet and bitter contrast, RTs were similar for the detection and discrimination tasks (*F*_(1,19)_ = 1.62, *p* = 0.219, η^2^ = 0.01), and RTs were faster for sweet than for bitter (*F*_(1,19)_ = 12.07, *p* = 0.003, η^2^ = 0.03). Accuracy was significantly higher in the discrimination than in the detection task (*F*_(1,19)_ = 7.10, *p* = 0.020, η^2^ = 0.09), and also higher for sweet than for bitter (*F*_(1,19)_ = 7.54, *p* = 0.010, η^2^ = 0.04). A task × taste interaction was observed (*F*_(1,19)_ = 8.67, *p* = 0.008, η^2^ = 0.07) and a comparison of the difference in accuracy between detection and discrimination revealed that the effect was larger for bitter than for sweet (*t*_(19)_ = −2.94, *p* = 0.008, *d* = 0.56).

### Classifier

Statistical analyses were performed on within-subject decoding results which are visualized as the grand-average performance in Figure 3*A*. Decoding onset times and the accuracy of the classifier, which was defined as the percentage of trials for which an onset was determined (i.e., at some point in time the taste was correctly identified for the predefined cluster period) are summarized in Table 2 and shown in Figure 3*C*. The contrasts separated the analyses for the taste pairs sour-salty and sweet-bitter in line with the study design as before. Because two participants performed poorly during the behavioral discrimination of salty and sour, too few trials remained for the decoder to learn their respective taste patterns. Hence, the analyses involving salty and sour tastes were computed on lower sample sizes (indicated by the lower number of degrees of freedom).

**Table 2.** Descriptive statistics of decoding onset times and accuracies

	Detection				Discrimination			
	Onset (ms)		Accuracy (%)		Onset (ms)		Accuracy (%)	
	M	SEM	M	SEM	M	SEM	M	SEM
Salty	136	12	92.2	1.2	304	18	66.8	5.1
Sour	147	11	95.4	1.1	285	25	61.7	4.7
Bitter	250	22	80.6	3.0	242	12	79.5	4.6
Sweet	245	17	80.8	3.1	242	15	80.0	4.7

For the salty and sour contrast, decoding onsets during detection were significantly faster than during discrimination (*F*_(1,17)_ = 44.75, *p* < 0.001, η^2^ = 0.53), and onset times were similar for both tastes (*F*_(1,17)_ = 0.16, *p* = 0.692, η^2^ = 0.001). Likewise, classifier accuracy was significantly higher during detection than discrimination (*F*_(1,17)_ = 35.01, *p* < 0.001, η^2^ = 0.50), and similar for both tastes (*F*_(1,17)_ = 0.87, *p* = 0.365, η^2^ = 0.001).

For the sweet and bitter contrast, decoding onsets were similar for both tasks (*F*_(1,19)_ = 0.13, *p* = 0.723, η^2^ = 0.001) and for both tastes (*F*_(1,19)_ = 0.04, *p* = 0.851, η^2^ = 0.00). Likewise, classifier accuracy did not differ among the tasks (*F*_(1,19)_ = 0.07, *p* = 0.794, η^2^ = 0.001) nor tastes (*F*_(1,19)_ = 0.03, *p* = 0.865, η^2^ = 0.00).

### Neural-behavioral correspondence

To verify the correspondence between the task-specific effects observed for decoding onsets and RTs, we calculated Pearson correlations of the taste- and subject-wise difference values between detection and discrimination latencies for decoding onsets and for RTs (Figure 3*D*). We observed significant positive correlations for salty (*r*_17_ = 0.40, *p* = 0.045), sweet (*r*_18_ = 0.57, *p* = 0.004), bitter (*r*_18_ = 0.47, *p* = 0.017), but no significant correlation for sour (*r*_17_ = 0.10, *p* = 0.343).

## Discussion

In this study, we investigated the processing sequence of simple and complex gustatory perceptual decisions, using electrophysiological patterns and behavioral responses elicited by salty, sour, sweet, and bitter tastants. Building on recent findings that taste category information is available within the time period of the earliest evoked response, we examined whether the detection and discrimination of a taste are simultaneous or distinct processing stages, and whether potential differences are represented early or late in the gustatory processing cascade. For the first time, we demonstrate not only a close correspondence between the earliest neural and behavioral responses, but also provide evidence that temporal differences between simple and complex taste-related decisions are established early during chemosensory encoding, rather than later during higher-level cognition. Interestingly, however, the latencies of detection and discrimination were contingent on the specific taste comparison, such that the temporal sequence varied with the hedonic contrast, suggesting that gustatory features may be processed partially in parallel.

For salty and sour, detection times were significantly faster than discrimination times, with a ∼100-ms difference in their neural onsets, and a 300- to 400-ms difference between behavioral responses, suggesting that gustatory features required for the mere detection and for taste category discrimination are processed sequentially so that the depth of processing increases with time. This observation is consistent with previous RT studies which showed that simple taste judgments such as taste detection are 100–200 ms faster than more complex judgments such as taste discrimination (Yamamoto and Kawamura, 1981; Halpern, 1986), and specifically that the discrimination of salty and sour requires even more time (an additional 400–600 ms) as compared to their individual taste detection (Kuznicki and Turner, 1986). The authors attributed this taste-specific increase in discrimination time to the failure of the time criterion strategy, which suggests that discrimination performance is controlled by the detection latency of the faster of two tastes which can be used as a response cue (essentially reducing the processing depth required for actual identification). Accordingly, the difference between taste detection and identification would be underestimated regularly, given that the speed at which a discrimination task is solved benefits from differing detection latencies between tastes, whereas discriminating tastes with similar detection latencies would reflect actual discrimination times. However, probing this hypothesis in gustation is not trivial because matching detection times are typically only observed for the juxtaposition of salty and sour.

In contrast to previous work, we observed no neural and only a minuscule behavioral difference in detection latencies for bitter and sweet, so that the likely failure of the time criterion strategy should have predicted an increase in discrimination time. Crucially, however, we observed similar processing times for the detection of sweet and bitter and their discrimination, both at the neural and behavioral level. The absence of any task dependency when comparing sweet and bitter suggests that a different mechanism, not available in the contrast of salty and sour, diminished the time lag between taste detection and discrimination. Thus, we argue that taste features that facilitate the identification process were available already early during taste processing, in line with the notion that the gustatory processing cascade does not simply constitute an invariant sequence of coding states (Fontanini and Katz, 2006, 2009; Samuelsen et al., 2012).

One apparent difference between the two taste-discrimination contrasts lies in the valence associated with the individual tastants. Whereas salty and sour were virtually identical with respect to their neutral hedonic value, sweet and bitter showed a marked difference, tending toward the positive and negative extremes of the pleasantness scale, respectively. While previous reports suggested that similar detection latencies caused the increase in discrimination times (Kuznicki and Turner, 1986), perhaps it was hedonic similarity that reduced stimulus distinctiveness instead. This would also be consistent with the comparably high error rates in the salty-sour discrimination and suggest that task difficulty increased concomitantly with processing times. Similar observations were made in olfaction, where discrimination of similar odors required additional processing time (Abraham et al., 2004). Likewise, for the sweet-bitter discrimination, valence may have served as the decisive response cue for the discrimination task, essentially substituting the presumed role of individual detection latency, and thereby compensating the need for additional processing time and potential performance impairments. Hence, the putative role of hedonics in taste identification emphasizes that the gustatory processing cascade unlikely unfolds in a purely serial manner but rather that taste detection, identification, and palatability are processed in parallel or with considerable overlap as it has been shown in rodents (Perez et al., 2013).

Anatomic and physiologic evidence from primates suggests that sensory and hedonic features of a taste event are indeed processed largely in parallel (Sewards and Sewards, 2002). In contrast, rodent studies revealed adaptations in the earliest taste response of amygdalar neurons to an aversive compared to a non-aversive taste, which further resulted in increased functional connectivity, implying greater information flow between amygdala and gustatory cortex (Grossman et al., 2008). Given adequate cross-talk within the gustatory network (cf. Katz et al., 2002), and given a faster conclusion of hedonic over chemosensory computations, the discrimination of any of two tastes could benefit from divergent hedonic information, thereby modifying the task to a recognition of taste palatability rather than category (or, alternatively, facilitating sensory identification itself). Evolutionarily, humans were likely to benefit from a taste system which commands a flexible coding mechanism with the capability to quickly incorporate hedonically relevant information. In fact, because the ultimate purpose of tasting is to determine whether an organism should ingest or reject a substance, it is only plausible to assume that this evaluative process relies considerably on hedonic evaluations, which may take precedence over sensory categorization or semantic retrieval. Therefore, the workings of the gustatory system appear to be related to what has been reported in the olfactory system (which largely coincides in its function to determine approach and avoidance), such that hedonic evaluations are processed in parallel to identification (Olofsson et al., 2013), and often precede odor naming (Lawless and Engen, 1977).

An alternative, although speculative, explanation of the taste-contrast specificity may be found in different taste transduction mechanisms starting in the peripheral gustatory system. Bitter and sweet taste are mediated by specialized, taste-specific GPCRs, which are expressed in distinct Type II taste receptor cells (Chandrashekar et al., 2006), and which converge on a common intracellular signaling pathway culminating in ATP release (Roper and Chaudhari, 2017). Interestingly, bitter compounds typically activate numerous bitter taste receptors, possibly to ensure detection of potentially toxic bitter-tasting substances via redundant activation (Meyerhof et al., 2010). Moreover, bitter and sweet are linked to specific behaviors: avoidance and approach, respectively. Hence, it is plausible to assume that the separation of sweet and bitter transduction pathways, along with differential encoding of palatability (whether the taste is pleasant or unpleasant), likely contribute to the superior discriminability of these two tastes, enabling their discrimination as soon as they are tasted.

Salty and sour, on the other hand, are mediated by specific ion-channels expressed in neuron-like Type III cells (Lewandowski et al., 2016). These are depolarized as a result of intra-cellular acidification for sour and possibly also for salty, and convey taste information via action potentials (Roper and Chaudhari, 2017), which may, at least in part, contribute to overall faster taste transduction (and faster resulting behavioral responses) compared to GPCR-mediated taste categories. Moreover, because taste-induced activations overlap for salty and sour, particularly at higher concentrations (Lewandowski et al., 2016), and because taste neurons are more broadly tuned with increasing concentrations (Wu et al., 2015), the downstream responses to these tastes may be somewhat more ambiguous and required additional processing to disentangle the sensory inputs, thereby increasing the processing time for the salty-sour discrimination. Of course, differences in the distribution of quality-specific receptor cells may have contributed to the present findings as well.

In conclusion, our results show a close correspondence between the patterns of taste-related psychomotor and the earliest electrophysiological responses, suggesting that behavioral effects are established early in the gustatory processing cascade during stages associated with chemosensory encoding rather than higher-level cognition such as decision making (Wallroth et al., 2018). While detection and discrimination of gustatory stimuli likely occur sequentially, hedonic computations which run in parallel to the purely sensory computations may facilitate taste identification. Hence, the gustatory processing cascade (including the perceptual stages or “milestones” of detection and discrimination) appears to be a variable sequence of sensory coding states contingent on the specific tastes and potentially other contextual factors.

## Synthesis

Reviewing Editor: Li Li, New York University Shanghai Decisions are customarily a result of the Reviewing Editor and the peer reviewers coming together and discussing their recommendations until a consensus is reached. When revisions are invited, a fact-based synthesis statement explaining their decision and outlining what is needed to prepare a revision will be listed below. The following reviewer(s) agreed to reveal their identity: Cody Cushing, Sidney A. Simon.

Both reviewers commented that the authors did a good job in revising the ms to address their concerns. There are however still some minor issues that need to be addressed before the ms is accepted for publication. I list their minor concerns below.

Reviewer 1

I would like to thank the others for the careful and thoughtful consideration and response to my previous comments. I find the manuscript much improved and my understanding of the work presented much clearer.

I appreciate the inclusion of topographical maps of eeg data. I do think the reader could benefit from a similar map around the time point detection and discrimination is occurring for sweet and bitter since there's little clarity here or in the literature (based on the authors own comments in their rebuttal) where the activity leading to these results is coming from, topographically speaking. I appreciate that localizing such activity is not the goal of the manuscript, so I'm fine without it. However, future researchers will be subject to the same level of uncertainty with regards to selecting relevant features/channels for similar analyses in future studies on the topic.

I'm happy to see that the results do not change much when excluding behaviorally incorrect trials or when switching to AUC to characterize classifier accuracy.

I would like to thank the authors for the much-improved clarity regarding the statistical analysis performed here. I do still have concerns regarding multiple comparisons but much less so now. The authors state in their rebuttal, “this procedure did not attempt to quantify all statistically significant periods (which would be the usual scenario of multiple comparisons) but only to identify the first such period within a trial to determine the taste-signal onset (i.e. the focus was when the effect occurs first)”, but this does not make the author's immune to the MCP inherent in testing data from multiple time-points. I understand the cluster adjustment procedure in place to control for this, but I still find this fairly liberal. I'd also like to note that the success/failure of a statistical test does not have any bearing whether a test is liberal/conservative. I'm not surprised at the number of trials for which an onset could not be determined for discrimination. The overall discrimination results shown in Fig. 3A are very weak. That being said, I sympathize with the exploratory nature of the study and why the authors would choose such a methodology. I would just like to see that commented on more in the manuscript since reader's will be unable to see the author's rebuttal to these concerns. The attempt to balance Type I and Type II errors in an exploratory study should be explicitly commented on.

These are mostly just discussion points which should hopefully be apparent to the discerning reader, so I'm happy see the manuscript move to publication pending these relatively minor revisions. Hopefully the line of research is continued either by the author's or other researchers and the robustness of the results can be determined by replication rather than the strictest statistical methods possible.

Minor notes:

Methods would benefit from a more explicit operationalized definition of onset determination. Presumably the first time-point for which a significant cluster is found on a single trial, but this should explicitly stated so that any future attempts for replication/extension are not vulnerable to a mistaken assumption

Please be explicit that all 64 electrodes are used in the analysis.

One too many '(' -line 240

Reviewer 2

use tastant rather than taste when referring to a chemical

also check number of significant digits both in p values and in data

## Author Response

Synthesis Statement for Author (Required):

Both reviewers think the ms is interesting but are not convinced by the results. Both think there is a serious leap of logic from data to conclusions and Reviewer 2 has serious concerns of the stats used. I list their detailed comments below:

Reply: We have included a point-by-point reply to the reviewers along with modified figures and text excerpts. Additionally, we have highlighted all major changes made to the manuscript with the exception of single words, deletions (are listed in the reply), and figure numbers.

Reviewer 1:

The authors present an EEG experiment looking at behavioral and neural results in response to a detection (sweet, sour, salty, and sweet versus a no-taste water control) and discrimination (salty vs. sour, sweet vs. bitter). The authors find that there is a significant behavioral RT latency difference in the detection and discrimination of salty and sour tastes, but no such difference for sweet and bitter tastes Similarly, using time-resolved decoding, they find identical differences (and lack thereof) in onset of significant decoding of detection and discrimination of these tastes. If valid, this would be an interesting finding. However, I find the statistical analysis ambiguous and inadequately defended against multiple comparisons as well as some other major methodological concerns?

1.) Why are no topogrophical maps of eeg data presented in any capacity? It is quite difficult to judge the EEG data quality based on one GFP plot for each condition. It'd be quite useful to see say a topographical plot of the EEG data at timepoints of significant decoder accuracy.

Reply: We have included topographical maps for each taste and task as the average from 150-200 ms in the revised Figure 2B. This time period represents well the first taste-evoked ERP positivity (P1); it's typical fronto-central positivity (see Ohla et al. 2012 for a review) is pronounced for salty and sour during the detection task. For sweet and bitter, which exhibit a later taste-evoked response, it is not yet observable at this time. We opted to present the maps for the same period for all tastes and tasks instead of tailoring the time periods to taste- and task-specific decoding or GFP onsets. We think this nicely represents the strong variability amongst tastes and obviously also tasks. Note that the maps are difference maps (taste minus water), which removes the visual portion of the evoked response that was elicited by the display of the fixation cross. The maps and procedures are now also described in the methods (p.7).

”To illustrate the electric field distributions, we computed topographical voltage maps for each taste and task. Each map represents the grand-averaged, mean voltage from 150-200 ms relative to water (Figure 2B). Difference maps were computed to remove the visual evoked response elicited by the display of the fixation cross.”

2.) Classifier Accuracy is a fine metric for decoding success, particularly when using an even number of trials in your analysis, which it seems from the manuscript you are. But you may consider still to use the area under the response operating characteristic curve as an alternative metric, as it tends to be more robust. This is more of a minor concern but it is related to my major concern below:

Reply: We have now computed the performance metric with the AUC. The results are displayed below in the revised Figure 3A (also excluding incorrect trials following the reviewer's subsequent concern #3). We initially chose accuracy instead of AUC because accuracy can be calculated per taste, thereby enabling us to display the performance for each taste independently (see original Fig 3A). Using the AUC, the performance can be displayed for the discrimination pairs only, but not for the individual tastes so that certain details of the classification are lost. For instance, during the discrimination between sweet and bitter the accuracy shows that the distinct signal of the bitter taste takes longer to emerge than for the sweet taste, whereas no such (albeit descriptive) inference can be made when visualizing the performance with the AUC.

Please note that this change does not affect the statistics presented in the paper because the performance metric is only relevant to the visualization of the grand-average performance in Figure 3A -- which is independent of the ANOVAs calculated on the (within-subject) single-trial onset times and percentage of trials with no discernable onset in Figure 3C.

3.) Despite that participants were largely accurate, accuracy did not really appear to be at ceiling for some of the conditions, notably discrimination of salty and sour. Yet, it seems like these incorrect trials were included in the procedure for decoding discrimination (methods say no trials were excluded)? I wouldn't think you could or would want to include incorrect trials in your classifier training, as you're essentially adding noise, which could harm say, the onset time of accurate decoding, which is a primary driving effect of the paper. I think incorrect trials should be excluded from these analyses and there's no way to determine whether the content that is attempted to be decoded from the neural data is actually present in it. Unbalanced trial counts are not ideal, but this is not as much of a concern when using the area under the ROC curve to quantify classifier accuracy. This is certainly preferable to using noise in your classifier for the sake of equal trial counts.

Reply: It is true that we did not exclude trials from training because we did not necessarily share the assumption that erroneous trials equal noise in the sense that the participants did not perceive the tastes (in which case such trials would indeed add noise to the pattern learning). Instead, we considered it just as probable that the participants might have had trouble solving the behavioral task (i.e. telling the tastes apart), while still clearly perceiving a taste sensation (in which case such trials can still represent taste-signal).

Despite this concern, we have recomputed the results after exclusion of all incorrect trials from training as suggested by the reviewer and present an updated Figure 3 (as above, but here next to the previous version for easy comparison). We observe that the results are largely identical, however for two participants during the salty and sour discrimination the decoder failed to extract taste patterns because too few trials remained for training after exclusion of incorrect trials (one participant incorrectly labelled salty as “sour” in 85% of discrimination trials, and another labelled sour as “salty” in 72.5% of discrimination trials). Accordingly, the ANOVAs on the salty and sour tastes (Fig. 3C) and the respective correlations in Figure 3D are computed on smaller sample sizes (from N = 20 to N = 18). We have updated all statistics and Figure 3 in the manuscript (using the right side option from the Figure below).

4.) I'm a bit confused on how the statistical analyses for the eeg data was performed:

Do you have a reference for the logic used in the decoding statistical analyses logic of cluster-adjusted binomial tests?

Reply: We apologize for the lack of clarity in the manuscript. To summarize, we applied a 'searchlight' over the probability outputs of a logistic regression classifier within single trials in clusters of 50 ms in the x-direction (training time) and 100 ms in the y-direction (testing/generalization time) thresholded by the binomial statistic. Essentially, we required a means to determine a taste-signal onset within individual trials (which are inherently noisy) as robustly as possible. To this end, we used a temporal clustering procedure in line with the “maximum cluster area” statistic (i.e. the number of members in a cluster which exceed a statistical threshold; Bullmore et al., 1999). We chose the binomial threshold as it is a well-established statistic to test classifier performance against chance level by adapting to the sample size (Combrisson et al., 2015), and in our scenario of testing at the single-trial level it synergized well with the probability outputs of the sigmoid function. The motivation for such an adjustment at the single-trial level is that the coherent occurrence of true signal is more likely than for noise (i.e. the probability of clustered signal exceeds that of clustered noise). The permutation-based adjustment procedure established by Maris and Oostenveld (2007) is potentially too strict for our purposes because its focus is to determine whether or not an effect is present rather than when exactly an effect occurs (cf. also Groppe et al., 2011). As such it is indeed a very useful tool to adjust for multiple comparisons during statistical testing if our focus was on whether tastes could be successfully decoded from EEG recordings. However, this has been demonstrated previously (cf. Crouzet et al., 2015), whereas the application in the present paper required a means to identify the taste-signal's onset as accurately as possible (i.e. with a minimal number of false alarms early during the epochs, and just as few misses of the onset of the true signal). Therefore, we implemented this more liberal yet ecologically valid temporal clustering procedure as a trade-off between type I and II error rates to specifically determine when the effect occurs. We have now tried to better justify the adjustment procedure in the manuscript (page 8-9):

“In order to determine the onset of the taste-signal at the single-trial level, we used a searchlight approach in line with the “maximum cluster area” statistic (i.e. a pre-defined number of neighboring time-points exceed a statistical threshold; cf. Bullmore et al., 1999). Given that the sigmoid function of the logistic regression naturally quantifies the certainty with which a classifier makes its decision, we defined a classification as accurate when the correct choice was made with a certainty exceeding the 95% confidence interval of the binomial threshold (a common statistic in classification analysis because it adapts the chance level to the sample size, cf. Combrisson & Jerbi, 2105). Because the decisional certainty is strongly affected by the hyperparameter C (the regularization constant), with negligible influence on the overall performance, we fixed the parameter at C = 0.005, which essentially shrinks the standard deviation of the normal distribution of decision values (as compared to the default of C = 1) for more robust onset estimations. The cluster size is a free parameter which was defined as 50 ms of a stable pattern average (x-direction) and 100 ms of 95% successful generalization (y-direction). This cluster-asymmetry reflects our prioritization of stable estimates at the single-trial level over average pattern stability. Classifier performance was summarized for grand-average visualization as the area under the receiver operating characteristic curve (AUC), and for the statistical analysis of the single-trial results the accuracy was defined as the percentage of trials for which an onset was determined successfully.”

5.) How does, “only regard[ing] correct classifications that appeared within a temporal cluster of 50 ms of a stable pattern average and 100 ms of 95% successful generalization” (lines 234-235), adjust for multiple comparisons? On what were these seemingly arbitrary decisions based? Is this to say that clusters were formed by looking at points for which a binomial test exceeded a p of 0.05? And then these clusters were considered significant if they extended high/wide enough in x,y domain of Fig. 3A? This seems an extremely liberal criterion, with no genuine correction for multiple comparisons. Indeed, the decoding accuracies presented seem mostly negligible. Most “significant clusters” seem to be at ~55% barely above chance.

Reply: Yes, the reviewer understands the adjustment procedure correctly. However, we want to point out that for the determination of the onsets (cf. Fig. 3C, left), this adjustment was performed at the single-trial level which makes it not quite as liberal as one may think: the number of trials for which no such (adjusted) onset could be found (cf. Fig. 3C, right) was considerable. Moreover, this procedure did not attempt to quantify all statistically significant periods (which would be the usual scenario of multiple comparisons) but only to identify the first such period within a trial to determine the taste-signal onset (i.e. the focus was when the effect occurs first).

The choice of parameters of a cluster test, for instance for the definition of spatial or temporal neighbors (here 50 ms training and 100 ms generalization time) are usually arbitrary (i.e. 45 ms and 105 ms, respectively, might be just as valid), which is a common complication for clustering procedures (cf. Groppe et al., 2011). Here, these values are simply the result of a rough estimation, such that too small of a temporal cluster requirement leads to unreasonably early onset times, whereas too large of a cluster requirement leads to a failure to detect an onset in the majority of trials. Again, we wish to note that this procedure was not introduced to adjust for multiple comparisons per se (because strictly speaking we did not perform a statistical test on the classifier performance), but rather to accurately determine the onset of the taste-signal within individual trials.

It is true that we did also apply this same adjustment procedure to the average performance plotted in Fig. 3A which would represent an actual instance of multiple testing, yet this figure merely serves to visualize the decoding performance and has no real bearing on the statistics of the paper. That is to say the grand-average performance was not used to determine when the effect occurred, and instead the ANOVAs were computed using the onset times which were determined at the single-trial level as described in the reply to the reviewer's comment #4. Here, the permutation adjustment from Maris and Oostenveld (2007) would have been a valid choice to determine the presence of an effect (irrespective of when), but we wanted to retain consistency throughout the methods and were not particularly concerned with the question if an effect occurs.

In summary, we understand the reviewer's concern of properly correcting for multiple statistical comparisons. We would like to emphasize that our aim was to reliably detect the first occurrence of the taste signal, rather than performing multiple statistical tests. The classification performance itself is comparable to other published work on human taste (Crouzet et al., 2015; Hardikar et al., 2018; Wallroth et al., 2018), and human smell (Jiang et al., 2017; Qu et al., 2016) perception. Notwithstanding, performance maximization was of minor concern in the present work.

6.) Moreover, salty and sour detection plots in Fig. 3A make it seem like decoding taste detection happens instantaneously, which doesn't quite seem to match the detection onset times presented in Fig. 3C. Are these not the same onset times?

Reply: Indeed these times are not identical. The plots in Fig. 3A represent the average decoding performance across 20 participants, whereas Fig. 3C displays the (across-trial) average onset time per participant (i.e. the boxplot represents N = 20 values). The apparent early onset of significant (grand-average) decoding performance in the detection of salty and sour tastes (Fig. 3A) is likely owed to a subset of participants who show a rapid response: within only 100 ms, six participants show an onset for salty detection, and four participants for sour detection. In this scenario, it would be misleading to look at the earliest time point in the significant grand-average cluster in Fig. 3A as the signal onset across participants (which represents a pooling of classification performance, i.e. if a small number of participants shows a rapid response, the grand-average underestimates when the signal occurs first across participants). The average within-subject onset time (i.e. the average of the N = 20 onset times, without any performance pooling) for the detection of salty and sour are 136 ms and 147 ms (now also included in Table 2), respectively (a timing which is in line with previous reports, cf. Crouzet et al., 2015). To summarize, we apologize for the confusion between what is displayed in Figure 3A and 3C and stress that all statistics were only derived from what is shown in Figure 3C, the values of which were obtained from single-trial analyses. We have now highlighted this in the manuscript as well (page 12):

“Statistical analyses were performed on within-subject decoding results which are visualized as the grand-average performance in Figure 3A. Decoding onset times and the accuracy of the classifier (...) are summarized in Table 2 and shown in Figure 3C. The contrasts separated the analyses for the taste pairs “sour - salty” and “sweet - bitter” in line with the study design as before. Because two participants performed poorly during the behavioral discrimination of salty and sour, too few trials remained for the decoder to learn their respective taste patterns. Hence, the analyses involving salty and sour tastes were computed on lower sample sizes (indicated by the lower number of degrees of freedom).”

7.) This kind of data seems quite appropriate for non-parametric cluster permutation tests like Maris & Oostenveld, 2007. why are these not used, which would provide actual type I control for clusters that would appear by chance compared to arbitrary thresholds?

Reply: We agree that this established method would have been a good choice if our focus was on whether tastes could be successfully decoded from EEG recordings (see Fig. 3A) - this has been shown already previously (Crouzet et al. 2015; Wallroth & Ohla, 2018; Hardikar et al. 2018). We were investigating, however, specifically the onset of the taste-signal obtained within single trials, and juxtaposed these effects with the behavioral findings. As such the ANOVAs were only applied to the data shown in Fig. 3B and 3C while no statistics were performed on the grand-average data (shown in Fig. 3A). As stated above in response to the reviewer's comments #4 and #5, our focus was when the effect first occurs, rather than if an effect occurs at all. In summary, we required a means of adjusting at the single-trial level to reliably detect the taste-signal onset that would be neither too liberal (leading to an abundance of early false alarms) nor too strict (leading to many trials in which the signal is missed).

Minor concerns:

It's not clear if all 64 EEG channels are used for decoding, but it seems that they are. This a very unfocal investigation. Is there not a subset of electrodes in an area related to gustatory processing that could be used?

Reply: Indeed, we decoded all 64 channels in the present paper. Generally speaking, it is true that EEG classification may benefit from feature extraction or reduction methods, of which the selection of a subset of electrodes based on prior knowledge is the most straightforward approach (for an overview, cf. Blankertz et al., 2011). However, for taste (much like smell), there were only a few reports so far that describe the topographical distributions of the electric field at the scalp over time. These reports show consistencies across studies, e.g. the P1 is typically restricted to frontal and fronto-central electrodes, but considerable across-subject variability and differences in experimental protocols (cf. Ohla et al, 2012) make a confident a priori selection of electrodes difficult.

In order to compare the decoding performance between our generalist approach (using the full set of 64 electrodes) and a more focal investigation, we have used a subset of seven frontal electrodes (AFz, Fz, FCz, F1, F2, FC1, FC2) based on theP1 topography, for which we recomputed the analysis (see Figure below). The pattern of performance is comparable between the two analyses, yet using the entire measurement space leads to significantly higher AUC scores (fig. 3A: AUCmax = 70.9%; fig 1B: AUCmax = 63.0%). Accordingly, a subset of electrodes seems to be sufficient to reproduce the findings. However, the selection of this subset naturally requires the exclusion of other channels and presents an experimenter bias as we know it from canonical ERP analyses, which represents an approach that we deem not appropriate, particularly, for the investigation of a sensory system of which so little is known as of yet.

Figure 1. Average within-subject decoding generalization across time for each of the tastes by task. Detection performance is obtained for the classification of a tastant against water (detection task trials); discrimination performance is obtained for the classification between two tastants (discrimination task trials). The diagonals of the matrices (identical training and testing time) correspond to the common decoding approach. The x-axis displays training times which represents the stability of an average taste pattern. The y-axis displays generalization or testing times which represents the emergence of the average pattern (x-axis) within individual trials. Warm colours reflect average performance increases as compared to chance level (50%), cold colours reflect decreases; black contour lines indicate statistical significance of the grand average as assessed via one-sided cluster-adjusted binomial tests (p &lt; .05). A) decoding performance (calculated as the area under the receiver operating characteristic curve) obtained using all 64 electrodes; B) decoding performance obtained using seven frontal electrodes (AFz, Fz, FCz, F1, F2, FC1, FC2).

Why is the upper frequency limit 6 hz vs 7 or 8 hz which are typically included in the theta range as the lower limit of the alpha range?

Reply: This frequency cut-off is based on recent findings showing that taste quality information is encoded within the power and phase information of the delta and lower theta frequency bands (roughly up to 6 Hz; Wallroth et al., 2018; Hardikar et al. 2018). We have included the reference in the revised manuscript (page 7):

“The frequency cut-off was based on recent findings showing that taste quality information is encoded within the power and phase information of the delta and lower theta frequency bands (roughly up to 6 Hz; Hardikar et al., 2018; Wallroth et al., 2018).”

References:

Blankertz, B., Lemm, S., Treder, M., Haufe, S., & Müller, K. R. (2011). Single-trial analysis and classification of ERP components--a tutorial. NeuroImage, 56(2), 814-825.

Bullmore, E. T., Suckling, J., Overmeyer, S., Rabe-Hesketh, S., Taylor, E., & Brammer, M. J. (1999). Global, voxel, and cluster tests, by theory and permutation, for a difference between two groups of structural MR images of the brain. IEEE transactions on medical imaging, 18(1), 32-42.

Combrisson, E., & Jerbi, K. (2015). Exceeding chance level by chance: The caveat of theoretical chance levels in brain signal classification and statistical assessment of decoding accuracy. Journal of neuroscience methods, 250, 126-136.

Crouzet, S. M., Busch, N. A., & Ohla, K. (2015). Taste quality decoding parallels taste sensations. Current Biology, 25(7), 890-896.

Groppe, D. M., Urbach, T. P., & Kutas, M. (2011). Mass univariate analysis of event‐related brain potentials/fields I: A critical tutorial review. Psychophysiology, 48(12), 1711-1725.

Hardikar, S., Wallroth, R., Villringer, A., & Ohla, K. (2018). Shorter-lived neural taste representations in obese compared to lean individuals. Scientific reports, 8(1), 11027.

Jiang, H., Schuele, S., Rosenow, J., Zelano, C., Parvizi, J., Tao, J. X., ... & Gottfried, J. A. (2017). Theta oscillations rapidly convey odor-specific content in human piriform cortex. Neuron, 94(1), 207-219.

Maris, E., & Oostenveld, R. (2007). Nonparametric statistical testing of EEG-and MEG-data. Journal of neuroscience methods, 164(1), 177-190.

Ohla, K., Busch, N. A., & Lundström, J. N. (2012). Time for taste--a review of the early cerebral processing of gustatory perception. Chemosensory perception, 5(1), 87-99.

Qu, L. P., Kahnt, T., Cole, S. M., & Gottfried, J. A. (2016). De novo emergence of odor category representations in the human brain. Journal of Neuroscience, 36(2), 468-478.

Wallroth, R., Höchenberger, R., & Ohla, K. (2018). Delta activity encodes taste information in the human brain. NeuroImage, 181, 471-479.

The paper has promise if these concerns can be adequately addressed. I find it well presented and discussed. Of course we just need to be sure the results presented justify the discussion.

Reviewer 2

In this paper the authors recorded EEG s while human subjects were given tastes on their anterior tongue of NaCl (salty), CA (acid) quinine ( Q-bitter) and Splenda (S-sweet). The goal was to determine if tastant identification and discrimination occurs in sequence or in parallel. Thus the tastants were paired to be iso- intense ( NaCl and CA) and Q and S, the latter two which differed in hedonic value. It appears that both sequential and parallel processing occur. I have some minor and some more serious issues that will be outlined below.

1.) One comment is that since they only recorded from the scalp and stimulated the tongue they really don't know what went on in between so discussion about this should be reduced.

Reply: We have reduced parts of the discussion as suggested in the comments below. Since there is very little related research in humans, we feel that we should not neglect the animal literature and the models derived from these findings though. We hope that reviewer agrees that our manuscript does not suggest that we test molecular or peripheral mechanisms but that we merely sought inspiration from those studies.

2.) Lines 48-52 can it be both labeled lines and across-fiber patterns - what difference does it make to your study?

Reply: We agree that the mechanism by which taste is tranduced does not immediately influence the interpretation of our results. We have, therefore, removed the reference:

“Each taste category is detected by specific receptors, mostly on the tongue (Roper and Chaudhari, 2017), and taste-specific information is transduced via segregated lines (Chen et al., 2011) and across-fiber patterns (Pfaffmann, 1959) to the brain stem, eventually arriving at dissociable cortical representations (Katz et al., 2002a; Schoenfeld et al., 2004; Pavao et al., 2014; Crouzet et al., 2015).”

3.) 56: Abraham and Carleton in an olfaction study showed that the reaction time for discriminating among odorants increases with their increasing similarities.

Reply: We have included the reference to Abraham et al. (2004; Neuron) in the discussion.

4.) 81: Katz and colleagues used IOCs to deliver tastants which is very different from how you applied the tastants and also when an animal is freely licking. The results from those studies are not applicable especially given the hedonic component is long over. Of course expectation may be a factor.

Reply: We are aware of that those studies used quite different means of stimulation. For that reason and also because taste processing in rodents is not identical to humans, we make no claim that their findings can be readily transferred to humans. Our statement “So far, our mechanistic understanding of the taste processing sequence is based on rodents, ... ” was merely to highlight that there is very little data on the dynamics of taste processing and this little data almost exclusively stems from rodents. In contrast to other senses, there is no recent data from non-human primates and no data at all from intracranial recordings in humans available to inspire human electrophysiological studies on taste. We find it therefore important to include the rodent literature despite the differences between species. To underline our intention and to avoid any misinterpretation, we have added the following to this section:

“So far, our mechanistic understanding of the taste processing sequence is based on rodents, where single neuron recordings in the gustatory cortex revealed separable stages of taste nonspecific action potential bursts, which likely represent oral somatosensation, and more complex, taste-specific responses (Katz et al., 2001; Baez-Santiago et al., 2016), although these findings cannot be readily transferred to humans given differences between species and experimental protocols.”

5.) 90-91: edibility would include valance.

Reply: The reviewer refers to our statement: “In olfaction, response-time data suggest a cascade with distinct processing stages for detection, identification, and edibility, which unfold in a causal, sequential manner, while valence computations may also run, at least in part, in parallel to identification (Olofsson et al., 2013).” We agree with the reviewer in that, intuitively, edibility would include valence. In their model, Olofsson et al. show that edibility is a process that requires identification of an odor, while valence can be assessed via a route parallel to and therefore without prior identification of an odor.

6.) 113-116 : Fill in { } .

Reply: This field was intentionally left open in accordance with the journal's guidelines to maintain anonymity of the authors. We would fill in the reference to the local ethics committee after acceptance.

7.) I am concerned about the fact that used supermarket salt which may not be pure NaCl and may contain impurities thus making the experiments hard to reproduce.

Reply: We understand the reviewer's concern. According to national regulations adhering to the CODEX STAN 150-1985 (Standard for food grade salt) “The content of NaCl shall not be less than 97% on a dry matter basis, exclusive of additives.”. Since we used salt without additives such as iodine or fluoride, the purity of the salt used here should be even higher and provide an adequate reference for replication. We have added the minimum purity of “&gt;97%” to the methods. Note that we will also add further details on the manufacturer, which we had left unspecified as local supermarket to warrant a “blind review”.

8.) I am also surprised that 5.2 mM CA is not aversive. Maybe it's the short length of time on the tongue. Also see line 352.

Reply: The reviewer is right -- ingesting a few milliliters of a 5.2 mM citric acid solution would be quite aversive. As the reviewer states, the amount and duration have an impact on perceived intensity. Here, the small amount (210 µL) combined with the short duration (900 ms) and immediately following rinse caused the citric acid sample to be well acceptable as is corroborated by the ratings.

9.) 118-134. It took me awhile to figure out what you actually did.

Reply: We have rephrased this section to increase clarity:

“Taste and rinse solutions were delivered with the GU002 gustometer (Burghart Messtechnik GmbH, Wedel, Germany), which stores solutions in separate bottles that each supply a syringe pump with a check valve (Iannilli et al., 2015). From there, solutions are transported via separate, 5 m long Teflon tubes to a manifold outlet where they mount together with compressed air to a spray nozzle that atomizes the liquids to an even spray. The spray nozzle is positioned 1-1.5 cm above the slightly extended tongue so that the spray covers a large area of the anterior, slightly extended tongue's surface. All tubes ran inside a hose filled with water at 38{degree sign}C until the manifold to keep the solutions at a constant temperature and to minimize any thermal sensations. During the experiment, the participant comfortably leaned against a forehead rest, which stabilized the head and held the spray nozzle in place. In this position, liquids were applied to the slightly extended tongue and not swallowed but collected in a bowl underneath the chin. The position was monitored online via camera to monitor positioning of the tongue and movements. The gustometer was set to apply regular, distinct spray pulses. During each pulse, 70 μl of liquid were dispensed during 100 ms; this period was followed by a pause of 200 ms, which served to separate consecutive spray pulses. Each taste stimulus consisted of three such pulses and amounted to a bolus of 210 μl delivered over a period of 900 ms (flow rate: 233 μl/s). The timing and flow rate were optimized to minimize mixing of individual spray pulses and to elicit the experience of a continuous flow of liquid to the tongue. The distinct spray pulses permit to embed a taste stimulus in the “flow” of control or water stimuli without tactile cue. Notably, participants experience a tactile “pulsing” only for a few seconds until the lingual somatosensory system is habituated.”

10.) 258: non-repulsive - meaning they did not spit them out ( I guess)

Reply: We used “non-repulsive” to describe that the samples were not unpleasant; being even remotely close to spitting would be unacceptable as such strong aversive reaction would introduce an unnecessary hedonic bias and skew the data. We have replaced the attribute with “acceptable” to make our intention clearer.

11.) 269- on: Please give actual numbers in the results section. In know some are in legends but this is results.

Reply: We have included the mean values with their standard errors for the behavioral data (Table 1) and decoding results (Table 2) in the results section.

12.) 365-389: reduce

Reply: We have shortened that paragraph, particularly the reference to rodent studies.

“Anatomical and physiological evidence from primates suggests that sensory and hedonic features of a taste event are indeed processed largely in parallel, it was previously deemed unlikely that hedonic representations support the discriminatory function of the sensory representations due to their anatomical segregation in subcortical structures and gustatory cortex (see Sewards and Sewards, 2002). In contrast, rodent studies revealed adaptations in the earliest taste response of amygdalar neurons to an aversive compared to a non-aversive taste, which further resulted in increased functional connectivity, implying greater information flow between amygdala and gustatory cortex (Grossman et al., 2008). Yet, neuronal populations in the rodent gustatory cortex also show overlapping subsets in the representation of taste concentration and palatability information (Sadacca et al., 2012), which gives reason for caution in the direct applicability of these findings to humans. Nonetheless, under the less stringent assumption of Given adequate cross-talk among the processing sites of within the gustatory network ... ”

13.) 390-412. The argument of ion channels and GPCRs is difficult for you to make as you only stimulated the anterior tongue and there could be a different receptor density that could account for the temporal differences. Note that visual processing which also uses GPCRs is very fast.

Reply: We understand the reviewer's concerns and now highlight in our revised manuscript that this explanation is speculative. We also mention the possibility that receptor density may play a role. Note that we do not argue that GPCRs are generally slow nor that they are sufficiently slow to cause the typical delay observed for sweet and bitter (100-300 ms compared to salty and spur). Rather we mention that, in gustation, transduction it appears that GPCRs and ion channels are expressed in different taste cell types that operate with different transduction mechanisms, ATP release and action potentials, respectively, that may account for time differences. Moreover, we discuss findings that suggest that additional differences in the coding further upstream that may contribute to an explanation as to why sour and salty are easy and fast to detect but hard and slow to discriminate, namely the ....... We agree that we cannot exclude that different receptor density (i.e. likely lower for bitter on the anterior half of the tongue) contributed to the findings, which we acknowledge now at the end of that paragraph (page 16):

“An alternative, though speculative, explanation of the taste-contrast specificity may be found in different taste transduction mechanisms starting in the peripheral gustatory system.”

“Of course, mere differences in the distribution of quality-specific receptor cells may have contributed to present findings as well.”

14.) Figure 2. Please explain why the first 100 or so ms is independent of ant tastant including water. You may have done that but I don't recall.

Reply: The GFP does not differentiate much between taste and water directly following time zero because, at this time, a visual event changes for all conditions: the fixation dot is replaced by a fixation cross to indicate the onset of the task-relevant (taste)event. This is described in detail in the procedures. The almost identical GFP very early during processing hence represents the visual portion of the ERP response. Note that the visual response is identical for all taste and water trials and would therefore not influence the statistical analyses. With the inclusion of topo maps, we have now also included a brief description of the visual portion contained in the evoked response (page 7):

“To illustrate the electric field distributions, we computed topographical voltage maps for each taste and task. Each map represents the grand-averaged, mean voltage from 150-200 ms relative to water (Figure 2B). Difference maps were computed to remove the visual evoked response elicited by the display of the fixation cross.”

15.) I like the honest way you presented B. Is it possible to show the results of a single trial?

Reply: Thank you. Figure 2B already displays ratings which were obtained as a single trial per taste after the experiment concluded.
